# The regulation and pharmacological modulation of immune complex induced type III IFN production by plasmacytoid dendritic cells

**DOI:** 10.1186/s13075-020-02186-z

**Published:** 2020-06-05

**Authors:** Karin Hjorton, Niklas Hagberg, Pascal Pucholt, Maija-Leena Eloranta, Lars Rönnblom

**Affiliations:** Department of Medical Sciences, Rheumatology, Science for Life Laboratory, Uppsala University, Rudbecklaboratoriet, Dag Hammarskjölds v 20, C11, 751 85 Uppsala, Sweden

**Keywords:** SLE, IFN, pDC, NK, HCQ, IRAK4

## Abstract

**Objective:**

Patients with systemic lupus erythematosus (SLE) have an ongoing interferon (IFN) production due to an activation of plasmacytoid dendritic cells (pDCs), which can be triggered to type I IFN synthesis by RNA containing immune complexes (RNA-IC). Considering emerging data suggesting a role of type III IFN in the SLE disease process, we asked if RNA-IC can induce type III IFN production in pDC and how this production can be regulated.

**Methods:**

Peripheral blood mononuclear cells (PBMCs) or immune cell subsets were isolated from healthy blood donors or SLE patients and stimulated with IC containing U1 snRNP and SLE-IgG (RNA-IC). Hydroxychloroquine (HCQ) and an interleukin receptor 1-associated kinase 4 inhibitor (IRAK4i) were added to cell cultures. Cytokine mRNA levels were determined with a microarray and protein levels with immunoassays. Single-cell RNA sequencing of pDCs using ddSEQ technology was performed.

**Results:**

Type III IFN mRNA and protein was induced in RNA-IC-stimulated pDC-NK and pDC-B cell co-cultures. A subset of activated pDCs (3%) expressed both type III and type I IFN mRNA. IFN-λ2, IFN-α2b, interleukin (IL)-3, IL-6, or granulocyte-macrophage colony-stimulating factor (GM-CSF) enhanced IFN-λ1/3 production 2–5-fold. HCQ and an IRAK4i blocked the RNA-IC-triggered IFN-λ1/3 production (*p* < 0.01). IFN-α2b and GM-CSF increased the proportion of SLE patients producing IFN-λ1/3 in response to RNA-IC from 11 to 33%.

**Conclusions:**

Type III IFN production is triggered by RNA-IC in pDCs in a TLR-MyD88-dependent manner, enhanced by NK and B cells as well as several pro-inflammatory cytokines. These results support a contributing role for both type I and type III IFNs in SLE, which needs to be considered when targeting the IFN system in this disease.

## Background

Systemic lupus erythematosus (SLE) is a systemic autoimmune disease characterized by the presence of autoantibodies and immune complex (IC) formation. Via Toll-like receptors (TLRs), ICs can trigger the activation of the interferon (IFN) system, an important feature of SLE [1, 2]. Hydroxychloroquine (HCQ), which interferes with endosomal TLR signaling, is the standard of care drug. A majority of SLE patients display an increased expression of IFN-regulated genes, an IFN signature, which contributes to the activation of immune cells, a sustained immune reaction, and several disease manifestations [3, 4]. The IFN system consists of three classes of proteins, IFN types I–III, with antiviral and immunomodulatory properties, acting on separate receptors with different expression profiles [5]. Type I IFNs are mainly responsible for the IFN signature, but both type II and type III IFNs can be measured in a proportion of SLE patients and could contribute to the IFN signature [6]. Despite this, the exact role of type III IFNs in the generation of the IFN signature and thus in the SLE disease process is, to a large extent, unknown.

The type III IFNs, which include IFN-λ1–3, or interleukin (IL)-29, IL-28A, IL-28B, respectively, and IFN-λ4, are the most recently discovered IFNs. Type III IFN signals via the heterodimeric IFN-λ receptor (IFNLR), and in contrast to the ubiquitously expressed IFN-α receptor (IFNAR), the IFNLR has a restricted expression, primarily to epithelial cells [7–9]. However, plasmacytoid dendritic cells (pDCs) as well as B cells, monocytes, and hepatocytes also express the IFNLR and can be targeted by IFN-λ [10]. Despite distinct receptors and different receptor expression patterns, type III and type I IFNs share a common downstream signaling pathway, including activation of STAT1–3 and IRF9 (ISGF3) recruitment. The gene expression induced by type III IFN is more limited than, but completely overlapping with, the type I IFN-stimulated genes [11, 12].

IFN-λ is produced by pDCs, antigen-presenting cells (APCs), and epithelial cells in response to viruses, bacteria, and fungi, through activation of TLRs and cytosolic DNA sensors [10, 13, 14]. Once activated, pDCs mainly produce type I IFNs, but previous studies have demonstrated that herpes simplex virus (HSV) induces IFN-λ production within a small subpopulation of the IFN-α producing pDCs [15].

One trigger of IFN-α production by pDCs in SLE is RNA or DNA containing IC, which via FcγRIIA-mediated internalization activate TLRs 7 or 9, respectively [16]. Furthermore, interaction of pDCs with other cell types affects their cytokine producing capacity, as illustrated by the enhanced cytokine synthesis by TLR-activated pDCs co-cultured with NK cells [17–19], which can be suppressed by monocytes from healthy individuals [18]. B and T cells also increase the IFN-α producing capacity of pDCs [20, 21]. Based on these findings, our group has developed a model for pDC activation in SLE, using RNA-IC as an inducer of type I IFN and other pro-inflammatory cytokines [22].

In the present study, we asked if RNA containing ICs (RNA-ICs) have the capacity to induce type III IFN production in pDCs from healthy blood donors and whether this production is confined to a subset of pDCs. Furthermore, we studied the influence of other pro-inflammatory cytokines on the type III IFN response, and if TLR inhibition, through the antimalarial drug hydroxychloroquine (HCQ) or an interleukin-1 receptor-associated kinase 4 inhibitor (IRAK4i), modifies the production of type III IFN. We finally investigated if immune cells from SLE patients produced type III IFN in reponse to RNA-IC exposure.

## Materials and methods

### Cell isolation and culture conditions

Peripheral blood mononuclear cells (PBMCs) were prepared by Ficoll-Hypaque (GE-Healthcare) density-gradient centrifugation of buffy coats from healthy blood donors (Dept. of Transfusion Medicine, Uppsala University Hospital) or of EDTA blood from SLE patients and their healthy controls. CD14-depleted PBMCs, pDCs, NK cells, and B cells were isolated from PBMCs by negative selection, using CD14 MicroBeads, pDC isolation kit II, NK Cell Isolation Kit, and B cell isolation kit II, respectively (Miltenyi Biotec). The cell purity was > 95% and viability 92–98% as determined by flow cytometry. CD14-depleted PBMCs (400 × 10^3^/well), pDCs (25 × 10^3^/well), NK cells (50 × 10^3^/well), or B cells (100 × 10^3^/well) were cultivated in a total volume of 100 μl/well, in flat-bottomed 96-well plates (Nunclon) at 37 °C with 5% CO_2_ as described previously [18]. PDCs (12.5 × 10^3^/well) and NK cells (25 × 10^3^/well) from SLE patients and their healthy controls were cultivated in a total volume of 50 μl/well in V-bottomed 96-well plates (Nunclon) [23]. When indicated, the cultures were supplemented with the following human recombinant cytokines: IL-3 (BioLegend), IL-6 (Roche), GM-CSF; Leukine (Beslex), IL-29, and IL-28A (Peprotech), IL-28B (Gibco), or IFN-α2b (IntronA; Schering Plough).

### Interferon inducers

IgG was isolated from 2 SLE patient sera containing autoantibodies to Sm and RNP by protein G chromatography [22]. The U1 snRNP particles [24–26], with a purity of minimum 90%, and SLE-IgG (RNA-IC) were used at final concentrations of 2.5 μg/ml and 1 mg/ml, respectively [18]. UV-inactivated herpes simplex virus type 1 (HSV) was used at a final concentration of 10% (v/v).

### Drugs

The small molecule drug IRAK4i I92 (ND-2158, Nimbus Discovery) [27] and HCQ (Sigma-Aldrich) were pre-titrated and used at final concentrations of 10 μM and 5.8 μM (pDCs and NK cells), or 10 μM and 7.8 μM (monocyte-depleted PBMCs), respectively. The cells were pre-incubated with I92 or HCQ for 30 min at 5% CO_2_, 37 °C before adding IFN-inducers [28].

### Immunoassays

IFN-λ1/3 was measured using an enzyme-linked immunosorbent assay (IL-29/IL28B DuoSet ELISA kit, R&D Systems). When priming with IFN-λ2, the acquired IFN-λ1/3 concentrations in cultures were adjusted, due to cross reactivity of the ELISA kit antibodies with IFN-λ2 [15]. This was done by subtracting the detected levels of IFN-λ1/3 in the IFN-λ2 supplemented medium, from the acquired levels in RNA-IC-stimulated and IFN-λ2-supplemented cell cultures (see additional file 1). Concentrations of IFN-λ2 were measured using IL-28A ELISA Kit (Abcam). IFN-α concentrations were measured by a dissociation-enhanced lanthanide fluoroimmunoassay (DELFIA), as previously described [29] (1 IU/ml corresponds to 5–10 pg/ml). All cytokine levels were determined after 20 h of cultivation.

### Gene expression microarray

mRNA levels in RNA-IC- or medium-stimulated cells from two healthy individuals were determined using the Agilent Whole Human Genome Microarray. Briefly, RNA was isolated after 6 h (Nucleospin RNA II, Macherey-Nagel) from pDCs, B cells, NK cells, and co-cultured pDC-NK or pDC-B cells. For further details, see additional file 2.

### Single-cell RNA expression profiling

PDCs from two healthy blood donors (50 × 10^3^cells/well) were stimulated with RNA-IC together with IFN-α2b (500 IU/ml) and IL-3 (1 ng/ml). Cell purity was > 95% as determined by flow cytometry staining of BDCA2. After 6 h, cells were pooled and washed with cold phosphate-buffered saline (PBS) and 0.1% human serum albumin. Cells were encapsulated in droplets and analyzed using the ddSEQ™ Single-Cell Isolator (Biorad) [30]. For further details, see additional file 2.

### Statistical analysis

Differences between two groups were analyzed with Wilcoxon signed rank test (paired samples) or Mann-Whitney test. Friedman’s test for multiple comparisons was used when analyzing differences between ≥ three groups (GraphPad Prism 8.0 software). *p* values ≤ 0.05 were considered significant, * refers to *p* < 0.05, **p < 0.01, ***p < 0.001, and ****p < 0.0001.

### Patients

All 23 patients fulfilled ≥ 4 of the 1982 American College of Rheumatology classification criteria for SLE [31]. Patients were 51 years old (median, range 32–81), with disease duration of 17.5 years (median, range 1–46). Disease activity was determined at the time of blood sampling using the SLEDAI-2 K [32]. Ten patients had a SLEDAI score between 1 and 10 whereas thirteen patients had SLEDAI score 0 (additional file 3). The local ethics committee approved the study protocol, and informed consent was obtained from all patients and controls.

## Results

### Type III IFN is induced in co-cultures of pDCs and NK cells or B cells stimulated with RNA-IC

**Fig. 1:**
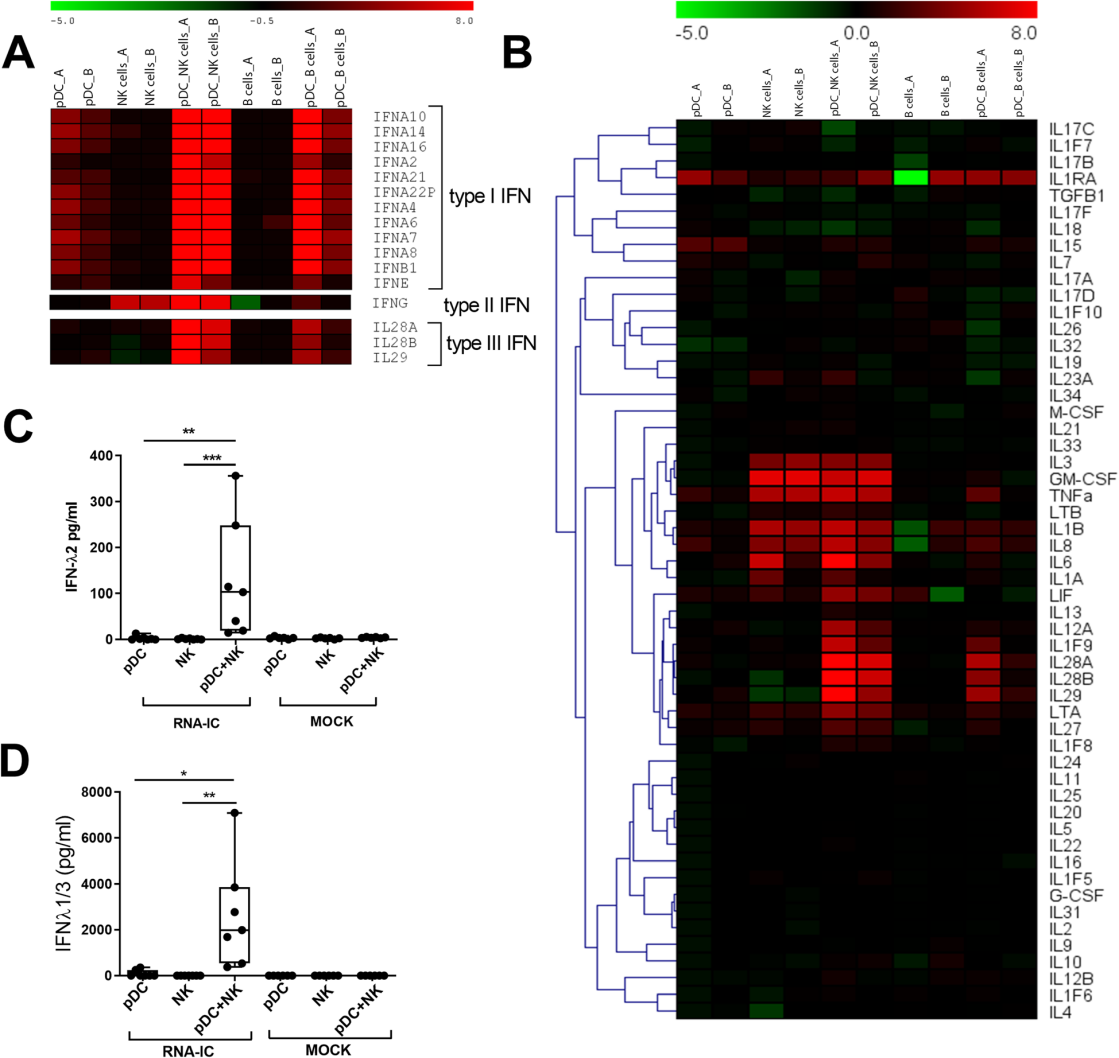
NK and B cells enhance the type III IFN production in pDCs stimulated with RNA-IC. **a**, **b** Relative signal intensity (log2fold change) of mRNA expression in RNA-IC-stimulated, vs mock-stimulated, cells from two healthy blood donors (**a** and **b**) after 6 h. Green indicates relative downregulation, black neutral, and red relative upregulation of gene expression. Protein levels of **c** IFN-λ2 and **d** IFN-λ1/3 in supernatants after 20-h stimulation. Boxplots show medians with interquartile range (seven donors, three independent experiments). Friedman’s test. *p < 0.05, **p < 0.01, ***p < 0.001

To clarify if RNA-ICs trigger the expression of type III IFN in immune cells from healthy blood donors, we performed a microarray analysis of RNA-IC-stimulated pDCs, NK cells, or B cells, as well as pDC-NK or pDC-B cell co-cultures. As can be seen in Fig. 1a, the co-cultures distinctly upregulated type III IFN mRNA in response to RNA-IC stimulation, more prominently in the pDC-NK co-cultures (> 200-fold) than in the pDC-B cells (> 15-fold). No relative increase in the expression of type III IFN genes was observed in RNA-IC-stimulated vs unstimulated pure pDC, NK, or B cell cultures. As expected, type I IFN was upregulated in RNA-IC-stimulated pure pDC cultures in addition to the pDC-NK or pDC-B co-cultures and type II IFN in NK and pDC-NK cell co-cultures. The pattern of substantial upregulation of gene expression in the co-cultures of pDC-NK or B cells was also noted for some other RNA-IC-induced pro-inflammatory cytokine genes, such as *IL12A* and *IL1F9* (IL-36) (Fig. 1b).

To determine if the increase in type III IFN mRNA expression corresponded with protein synthesis, cytokine levels in supernatants were quantified. As shown in Fig. 1c, d, RNA-IC induced no or low amounts of type III IFN in pDCs (IFN-λ2; median 0 pg/ml, range 0–13 pg/ml, IFN-λ1/3; median 86 pg/ml, range 0–354 pg/ml) and NK cells (median and range 0 pg/ml), whereas the levels were much higher in the pDC-NK cell co-cultures (median 103 pg/ml, range 15–357 pg/ml, and median 1988 pg/ml, range 377–6720 pg/ml, for IFN-λ2 and IFN-λ1/3, respectively). Also in pDC-B cell co-cultures, IFN-λ1/3 levels were elevated compared to pure pDCs (additional file 4). Observing a more prominent increase in the type III IFN expression by pDC-NK cells compared to pDC-B cell co-cultures, we proceeded to focus on the co-cultures of pDCs and NK cells. Furthermore, and in line with the microarray data, IFN-α levels in the pDC-NK and pDC-B cell co-cultures were significantly elevated compared to pure pDC cultures (additional file 5).

Thus, IFNs are produced in response to RNA-IC, and type I and III IFN production is increased by co-cultivation of pDCs with NK cells or B cells.

### Type III IFN and type I IFN promote the production of type III IFN

**Fig. 2:**
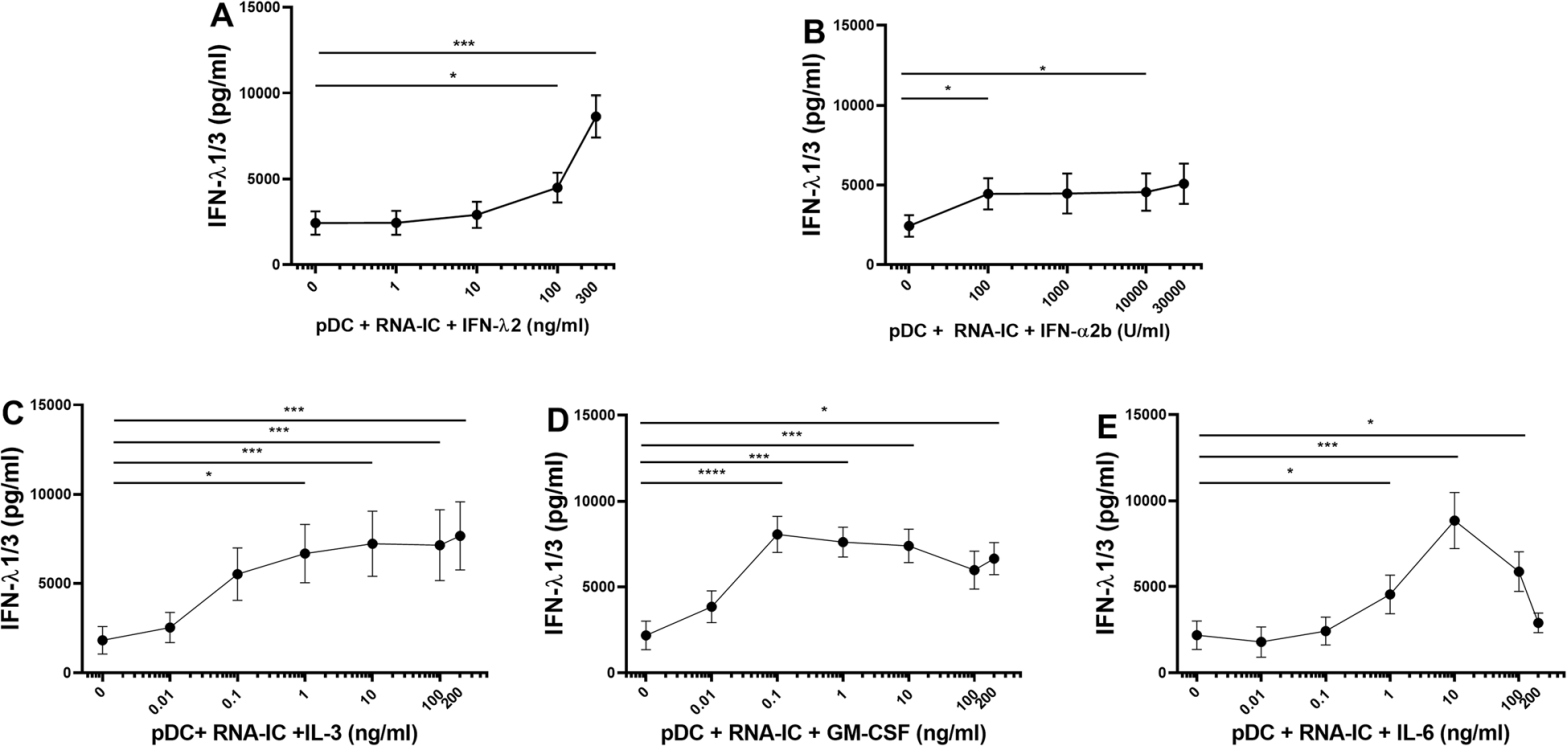
IFN-λ2, IFN-α, IL-3, GM-CSF, and IL-6 increase IFN-λ1/3 production by pDCs stimulated with RNA-IC. The levels of IFN-λ1/3 in supernatants from healthy donor pDCs stimulated with RNA-IC for 20 h in the presence of **a** IFN- λ2, **b** IFN-α2b, **c** IL-3, **d** GM-CSF, or **e** IL-6 in indicated concentrations. No IFN production was detected in the absence of RNA-IC. **a** Cross reactivity of IFN-λ2 in the IFN-λ1/3 immunoassay was corrected for as described in additional file 1. Graphs show means with SEM based on 5–6 donors in two independent experiments per cytokine. Friedman’s test. *p < 0.05, **p < 0.01, ***p < 0.0005, ****p < 0.0001

Next, we asked if type III IFN itself can enhance the IFN-λ1/3 production by RNA-IC-stimulated healthy blood donor pDCs, similar to the stimulating effect of type I IFN on the IFN-α production by pDCs, also termed priming [18, 33]. Furthermore, we asked if IFN-α exerts a priming effect on IFN-λ production. Figure 2a shows that IFN-λ2 exhibits a significant priming effect on IFN-λ1/3 production with a maximum 5-fold increase, whereas a 2-fold priming effect by IFN-α2b was observed (Fig. 2b).

Given the priming effect by IFN-λ2, we investigated if the RNA-IC-induced type III IFN synthesis by pDCs can be enhanced by other cytokines besides the type I and III IFNs. As seen in Fig. 2c–e, the IFN-λ1/3 production by RNA-IC-stimulated pDCs was significantly enhanced already at 0.1 ng/ml by IL-3 and GM-CSF and at 1 ng/ml by IL-6 to maximum 4-fold levels. Likewise, IFN-α production was enhanced by IL-3, GM-CSF, and IL-6 (additional file 6). Levels of RNA-IC-induced IFN-α were 30–40 fold higher than corresponding IFN-λ1/3-levels, and the relative increase in IFN-α was greater than that in IFN-λ1/3 (8–10-fold).

**Fig. 3:**
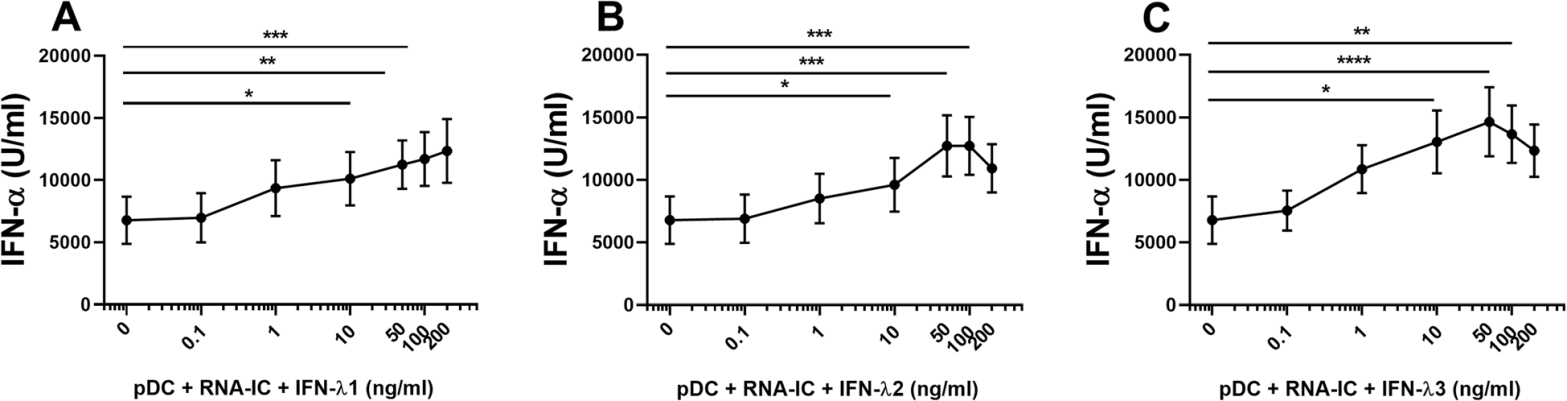
Type III IFNs increase IFN-α production by pDCs stimulated with RNA-IC. The levels of IFN-α in supernatants from healthy donor pDCs stimulated with RNA-IC for 20 h in the presence of **a** IFN-λ1, **b** IFN-λ2, or **c** IFN-λ3 at indicated concentrations. No IFN production was detected in the absence of RNA-IC. Graphs show means with SEM based on six donors in two independent experiments. Friedman’s test. *p < 0.05, **p < 0.01, ***p < 0.001

We also investigated whether type III IFNs affected the RNA-IC-stimulated IFN-α production by pDCs. Figure 3 shows that IFNs-λ1–3 increased the IFN-α levels to maximum 2-fold at a concentration interval of 10–100 ng/ml. IL-1β, MIP-1β, and TNF-α added to cell cultures at concentrations 0–10 ng/ml did not affect the RNA-IC-induced IFN-λ1/3 or IFN-α production (data not shown).

In summary, both type III and type I IFNs as well as IL-3, IL-6, and GM-CSF enhance the production of type III and type I IFNs by RNA-IC-stimulated pDCs. The pDCs display a more pronounced IFN type I than type III response to RNA-IC stimulation and to priming.

### A small subset of pDCs produce both type I and type III IFNs in response to RNA-IC

**Fig. 4:**
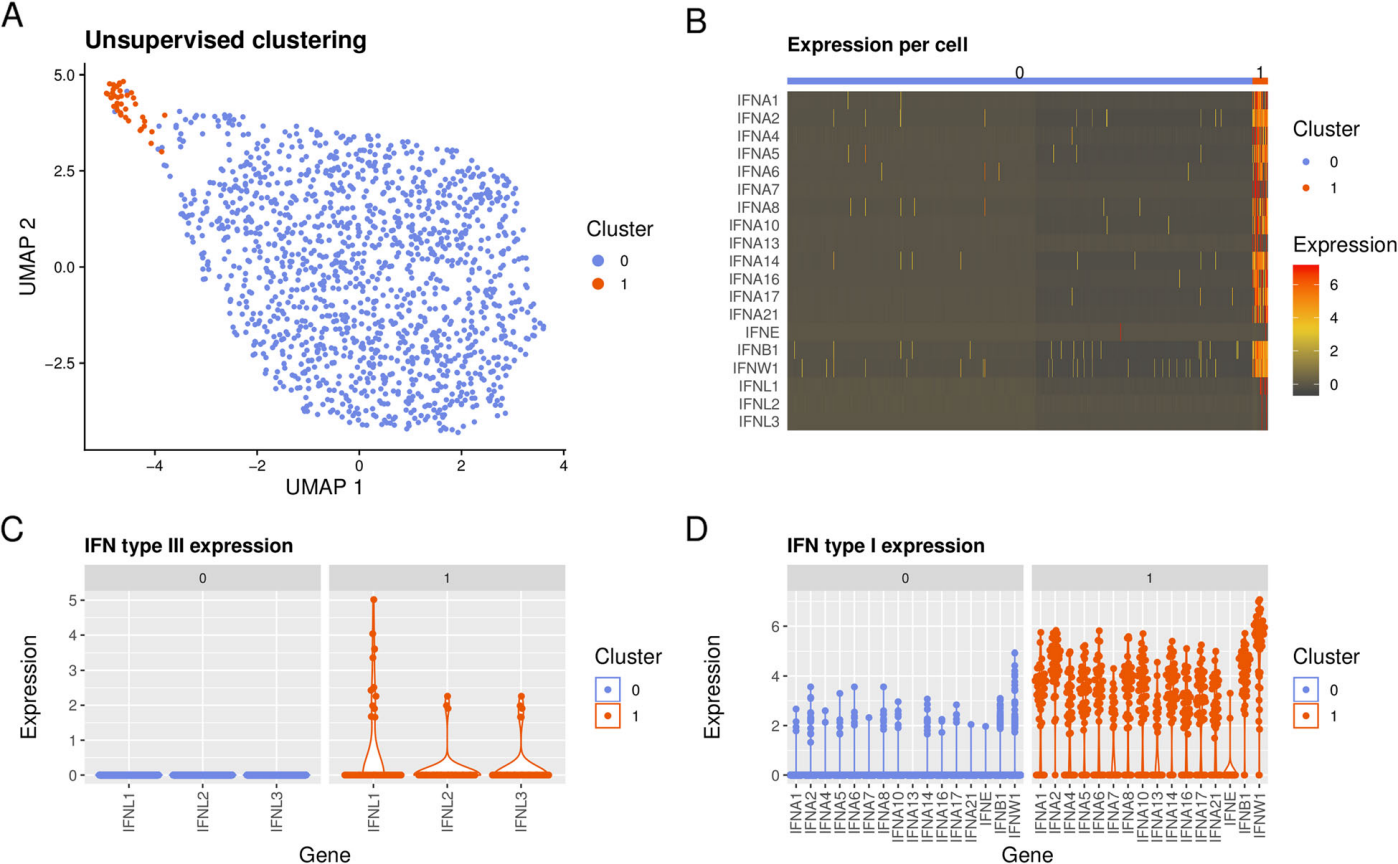
Type I and type III IFN expression in pDCs on the single-cell level. **a** Results from single-cell RNA sequencing illustrated by unsupervised clustering of 1413 healthy blood donor (*n* = 2) pDCs by non-linear two-dimensional Uniform Manifold Approximation and Projection (UMAP) embedding. Cells were stimulated with RNA-IC, IL-3, and IFN-α2b. Cluster “0” (blue) and cluster “1” (orange). **b** IFN gene expression per cell for cluster “0” and “1”. Individual cell expression levels of subtypes of **c** type III IFNs, and **d** type I IFNs, within clusters “1” and “0”. The cell purity was > 95% as determined by flow cytometry staining of BDCA2

To clarify if different subclasses of pDCs account for the production of type I and type III IFNs, pDCs from healthy blood donors were stimulated with RNA-IC and single-cell RNA sequencing was performed. Unsupervised clustering using the 2000 most variable genes identified two distinct clusters of pDCs (Fig. 4a), of which the minor subset, cluster 1 (3.2%), was essentially characterized by IFN gene activation (Fig. 4b). In total, 164 differentially expressed genes (DEGs) (adjusted *p* value < 0.05) were identified between the clusters. Type III IFN, dominated by IFN-λ1, was exclusively expressed in cluster 1 (Fig. 4c). Moreover, type I IFN genes were induced in the majority of cells in cluster 1 and at higher levels compared to cluster 0, where a minority of cells expressed low levels of type I IFNs (Fig. 4d). When comparing the most significantly differentially expressed genes between cluster 1 and cluster 0 (adjusted *p* value < 1 × 10^−15^, *n* = 54), besides the IFN gene predominance in cluster 1, a significant upregulation of other genes mapping to immune activation was found, such as cytokine and chemokine genes *CCL4*, *CCL3*, *TNF*, *CCL3L3*, and *IL12A* (log2FC > 1) as well as *CD40* (additional file 7). In cluster 0, on the other hand, 19 genes were overexpressed compared to cluster 1 (of which four exceeded log2FC > 1, additional file 8). Among these, *TLR7*, *STAT1*, and *OAS1* were noted, as well as several ribosomal protein genes.

Hence, a small minority of pDCs are responsible for the upregulated IFN gene expression upon RNA-IC stimulation, and type III IFN gene expression occurred within a subset of the type I IFN expressing pDC population.

### Type III IFN production in RNA-IC-stimulated pDC and pDC-NK co-cultures is inhibited by an IRAK4 inhibitor and by hydroxychloroquine

**Fig. 5:**
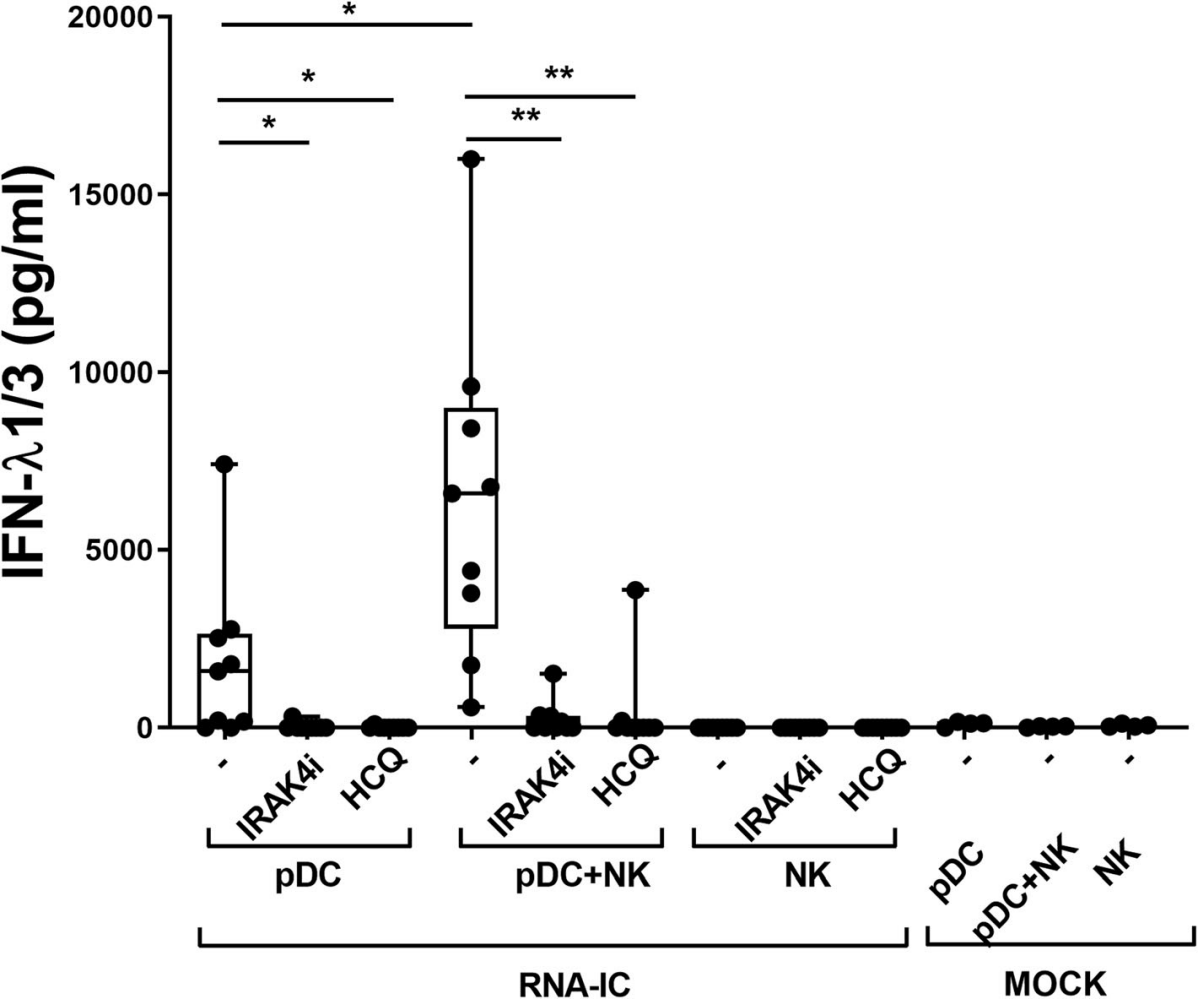
IFN-λ1/3 production by pDCs and pDC-NK cell co-cultures is inhibited by an IRAK4i and HCQ. The levels of IFN-λ1/3 after 20 h in supernatants from healthy donor PDCs and NK cells, stimulated with RNA containing immune complexes (RNA-IC) with or without IRAK4i (10 μM) or HCQ (5.8 μM). Boxplots show medians with interquartile range, based on nine donors in three independent experiments. Friedman’s test. *p < 0.05, **p < 0.01

Considering that IFN induction by RNA-IC is mediated through endosomal TLR binding, we asked if HCQ could inhibit this production. In RNA-IC-stimulated pDCs and NK cells from healthy blood donors, HCQ almost completely blocked the IFN-λ1/3 production (Fig. 5). An IRAK4 inhibitor, which acts downstream of the endosomal TLRs, was equally effective in blocking the IFN-λ1/3 production by the pDCs and pDC-NK cell cultures.

Thus, RNA-IC-induced type III IFN production by pDCs and NK cells is inhibited by HCQ as well as by an IRAK4 inhibitor.

### RNA-IC induces type III IFN production in immune cells from a subset of SLE patients, and GM-CSF and IFN-α2b increase the proportion of responders

**Fig. 6:**
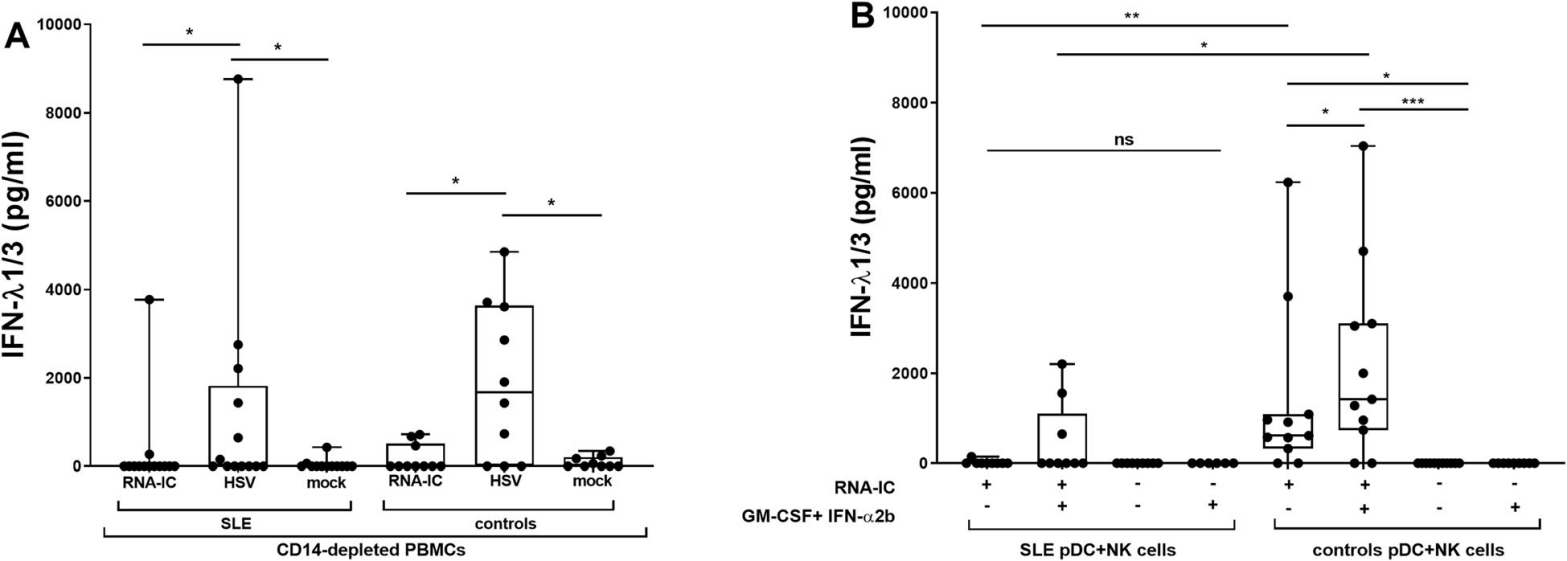
IFN-λ1/3 production is triggered by RNA-IC in immune cells from SLE patients and healthy controls. The levels of IFN-λ1/3 in supernatants after 20 h in **a** monocyte-depleted peripheral blood mononuclear cells (PBMCs) stimulated with RNA-IC or herpes simplex virus (HSV) or **b** plasmacytoid dendritic cell (pDC)-NK cell co-cultures with RNA-IC in the presence or absence of granulocyte-macrophage colony-stimulating factor (GM-CSF) and IFN-α2b. Boxplots show medians with interquartile range, based on **a** 13 patients and 10 healthy controls or **b** 9 patients and 11 healthy controls. Mann-Whitney test (SLE vs controls), Wilcoxon’s matched pairs signed rank test (RNA-IC vs HSV, priming vs no priming). *p < 0.05, **p < 0.01

We finally investigated cells from SLE patients and compared their IFN production with cells from healthy controls. In monocyte-depleted RNA-IC-stimulated PBMCs, IFN-λ1/3 was detected in 15% (*n* = 2) and 30% (*n* = 3) of patients and controls, respectively (Fig. 6). In comparison, higher IFN-λ1/3 levels and larger proportions of patients and controls producing IFN-λ1/3 were observed in response to the TLR 9 agonist HSV1. In purified, RNA-IC-stimulated, pDC and NK cell co-cultures from SLE patients, IFN-α2b and GM-CSF priming marginally increased the IFN-λ1/3 levels (from means 14 to 455 pg/ml, *p* = 0.125) and the proportion of responders (from 11 to 33%, *n* = 1–3). RNA-IC-induced IFN-α levels were higher than IFN-λ1/3 levels and detected in a larger proportion of patients and controls (additional file 9). PDC-NK cell co-cultures from all IFN-λ1/3 producing individuals responded to RNA-IC with IFN-α production. IFN production was lower in SLE patients than in healthy controls.

In conclusion, type III IFN production by monocyte-depleted PBMCs and pDC-NK cell co-cultures can be induced by RNA containing IC, in a subset of SLE patients. A trend toward higher IFN-λ1/3 levels, as well as higher numbers of RNA-IC responders, was observed after IFN-α2b/GM-CSF treatment.

## Discussion

In this report, we show that nucleic acid containing ICs induce type III IFN expression and protein production in pDCs from healthy blood donors. Using single-cell RNA sequencing, we also show that type III IFN expression in response to RNA-IC occurs within the small pDC subset that produces type I IFN, which is in line with previous observations [15]. These findings connect the type I and type III IFN production in pDCs and suggest that similar mechanisms may be operative in pDCs for the production of both these cytokines. Furthermore, since RNA containing ICs are present in SLE and can activate pDCs, our observations could be relevant to the understanding of the observed presence of type III IFN in serum and tissues in a subset of SLE patients [18, 22, 34].

The significant increase of type III IFN production noted, when NK or B cells were added to healthy pDC cultures, shows that type III IFN synthesis can be regulated by other immune cells. This observation indicates that the type III IFN response is part of the cross talk between different immune cells in the presence of nucleic acid containing ICs, as in SLE. Such cell interactions have previously been shown to contribute to the type I IFN production by pDCs [18–21]. The most powerful effect has been observed by NK cells, mainly mediated through cell-cell contact via lymphocyte function-associated antigen (LFA)-1 [19]. Herein, we also demonstrate that cytokines, previously shown to be increased in the serum or tissue of patients with SLE, strongly enhance the type III IFN production by healthy pDCs. These cytokines include IFN-α [35], GM-CSF [21, 36, 37], IL-6 [38, 39], and IL-3 [40–42], but also type III IFN itself [6, 43]. Furthermore, all type III IFN subtypes enhanced the IFN-α production. Taken together, these observations indicate that type III IFNs could partake in the positive feed-forward loop seen in SLE and contribute to the vicious circle with an ongoing IFN production and sustained immune activation [44].

The impact of type III IFN on the IFN signature in SLE probably varies between patients, as only a proportion of patients responded to RNA-IC and the magnitude of the type III IFN production differs considerably between individuals. A great inter-individual variation of the IFN-α response has previously been described, its production being affected by a number of genes [45], and it is possible that genetic variation affects the type III IFN response as well. The selection of cells investigated for gene expression profile (i.e., the IFN signature) is also of importance, as the IFNLR receptor is expressed by a limited number of cell types [8, 10]. The fact that pDCs displayed a more pronounced IFN type I than type III response to RNA-IC stimulation and to priming, however, supports the notion that type I IFN is more important for the IFN signature in SLE.

Both HCQ and an IRAK4 inhibitor blocked the type III IFN production by pDCs and NK cell co-cultures in response to RNA-IC stimulation. Thus, the production triggered via the TLR7-MyD88 signaling pathway can be inhibited by drugs targeting a pro-inflammatory route considered important in SLE. Whether HCQ or IRAK4 inhibition also can block type III IFN production in other cell types than pDCs remains to be determined. This is important, since expression of type III IFN to a large extent has been shown to occur at mucosal sites and in tissues, where it is produced by a number of different cell types. In fact, one major effect of type III IFNs is promoting the local immunity and preventing microbiologic invasions, rather than affecting systemic inflammation [10, 13]. Pursuing to block the potentially deleterious type III IFN production in SLE patients may therefore harbor certain risks, as it is conceivable that a complete inhibition may cause an increased propensity for infections, especially via the mucosal tissues. On the other hand, specifically blocking the type I IFN response may preserve the mucosal antiviral defense system, yet perhaps only partially ameliorate SLE disease manifestations [46].

There are several observations suggesting that type III IFN is of importance in SLE. Thus, IFN-λ1 can be detected in SLE patients’ blood as well as in lupus nephritis crescents and cutaneous lupus erythematosus (CLE) skin lesions [43, 47]. Serum levels of IFN-λ1 correlate with disease activity and specific organ manifestations, such as arthritis and nephritis, as well as the presence of antibodies to double-stranded DNA [48, 49]. Furthermore, serum IFN-λ3 levels have been associated with active mucocutaneous lupus disease [50]. Genetic polymorphisms in IFN-λ3/4 have also been associated with lupus nephritis susceptibility, indicating a role for type III IFN in active SLE [49]. Given these observations, we proceeded to investigate if immune cells from patients with SLE can produce type III IFN.

We noted that RNA-ICs triggered IFN-λ1/3 production in monocyte-depleted PBMCs from a minority of patients and the proportion of responding individuals was lower than in healthy controls. In the co-cultivated purified pDCs and NK cells from SLE patients, only a small subset of patients responded to RNA-IC stimulation with type III IFN production. There are several possible reasons for this observation, for instance reduced IFN producing capacity of patients’ pDCs, due to treatment, concurrent infections, or a refractory state following in vivo activation [51]. Furthermore, the amount of produced type III IFN was markedly lower than that of type I IFN, which perhaps is not surprising when considering that only a subset of the IFN-α expressing pDCs expressed IFN-λ1/3 in the single-cell RNA seq. A modest priming effect by GM-CSF and IFN-α2b was observed, increasing the number of type III IFN producing individuals and the levels of type I IFN production in SLE patients. This may be of relevance for the in vivo situation in SLE with increased levels of several pro-inflammatory cytokines, as discussed above. However, large patient cohorts followed longitudinally are needed in order to clarify the connection in SLE between type III IFN production and disease activity, as well as the impact of HCQ treatment.

There are limitations to this study. When isolating subsets of immune cells from SLE patients, the availability of blood limits the experiment size and numbers. In addition, migration of pDCs to tissues makes it difficult to obtain a sizable number of these cells for more extensive experiments [52]. It also cannot be ruled out that the IFN-λ1/3 response to RNA-IC in our samples is somewhat underestimated, due to the relatively high lower level of quantification (LLoQ 62.5 pg/ml) for the applied IFN-λ1/3 ELISA. Furthermore, differences in SLE activity and medication at the time of blood sampling could affect the results. The current study, however, consisted mostly of patients with low disease activity and no drug effect was evident. Despite these limitations, we believe that the collective data presented in this study identify one likely mechanism for type III IFN induction in SLE.

## Conclusions

In conclusion, we have shown that RNA-ICs activate a subset of pDCs to type III IFN synthesis and that this type III IFN production is promoted by other immune cells and several cytokines. We have also shown that targeting the TLR-MyD88 pathway inhibits the type III IFN production. As emerging data suggests that increased production of type III IFN is important in the SLE disease process, we propose that this IFN needs to be considered when developing drugs aiming to block or modulate the IFN system in SLE.

## Supplementary information

**Additional file 1: Figure S1.** Interferon (IFN)-λ2 displays cross reactivity in immunoassays of Interferon (IFN)-λ1/3, but not IFN-α.

**Additional file 2: Methods.** Gene expression microarray. Single-cell RNA expression profiling

**Additional file 3: Table S1.** Clinical characteristics of SLE patients and their treatment at the time of blood sampling.

**Additional file 4: Figure S2.** Type III IFN production is induced in pDCs and pDC-B cell co-cultures stimulated with RNA containing immune complexes (RNA-IC).

**Additional file 5: Figure S3.** Type I IFN production is induced in pDCs and pDC-NK or pDC-B cell co-cultures stimulated with RNA-IC.

**Additional file 6: Figure S4.** Interleukin (IL)-3, IL-6, GM-CSF and interferon (IFN) –α increase type I IFN production by pDCs stimulated with RNA-IC.

**Additional file 7: Table S2.** Differentially expressed genes (DEGs) in RNA-IC stimulated pDCs overexpressed in cluster “1” vs cluster “0”. 54 top overexpressed genes in cluster “1” compared to cluster “0”.

**Additional file 8: Table S3.** Differentially expressed genes (DEGs) in RNA-IC stimulated pDCs overexpressed in cluster “0” vs cluster “1” following unsupervised clustering of the 2000 most variable genes.

**Additional file 9: Figure S5.** Interferon (IFN)-α production is triggered by RNA containing immune complexes (RNA-IC) in immune cells from systemic lupus erythematosus patients (SLE) patients and healthy controls.

## Data Availability

The gene expression microarray datasets and the processed single-cell RNA seq data are available in Gene Expression Omnibus (GEO) (accession number GSE149456). The single-cell RNA seq raw data are available upon request from the authors on a collaborative basis and will be made available through a central repository when data safety regulations permit. All other data analyzed during this study are included in this published article and its supplementary information files.

## Abbreviations

*BDCA*: Blood dendritic cell antigen
*CCL*: C-C motif chemokine ligand
CD: Cluster of differentiation
DELFIA: Dissociation-enhanced lanthanide fluorescence immunoassay
FcγRIIA: Fragment crystallizable receptor IIA
FDR: False discovery rate
FITC: Fluorescein isothiocyanate
GM-CSF: Granulocyte-macrophage colony-stimulating factor
HCQ: Hydroxychloroquine
IC: Immune complex
IFN: Interferon
*IFNA2*: Interferon alpha 2
Ig: Immunoglobulin
IL: Interleukin
IRAK: Interleukin-1 receptor-associated kinase
IRAK4i: IRAK4 inhibitor
*IRF*: Interferon regulatory factor
ISGF: Interferon-stimulated gene factor
mAb: Monoclonal antibody
MIP: Macrophage inflammatory protein
MyD88: Myeloid differentiation primary response protein 88
NK: Natural killer
PBMC: Peripheral blood mononuclear cell
pDC: Plasmacytoid dendritic cell
RNA-IC: Ribonucleic acid containing immune complex
RNP: Ribonucleoprotein
SLE: Systemic lupus erythematosus
Sm: Smith nuclear antigen
snRNP: Small nuclear ribonucleoprotein
*STAT*: Signal transducer and activator of transcription
TLR: Toll-like receptor
TNF: Tumor necrosis factor
