# Supplementary material for: The regulation and pharmacological modulation of immune complex induced type III IFN production by plasmacytoid dendritic cells
Source: Arthritis Res Ther. 2020 Jun 5;22:130. doi: 10.1186/s13075-020-02186-z (PMC7275601; doi:10.1186/s13075-020-02186-z)
Supplement: Supplementary file 1 — Additional file 1: Figure S1. Interferon (IFN)-λ2 displays cross reactivity in immunoassays of Interferon (IFN)-λ1/3, but not IFN-α. [file 13075_2020_2186_MOESM1_ESM.pdf]

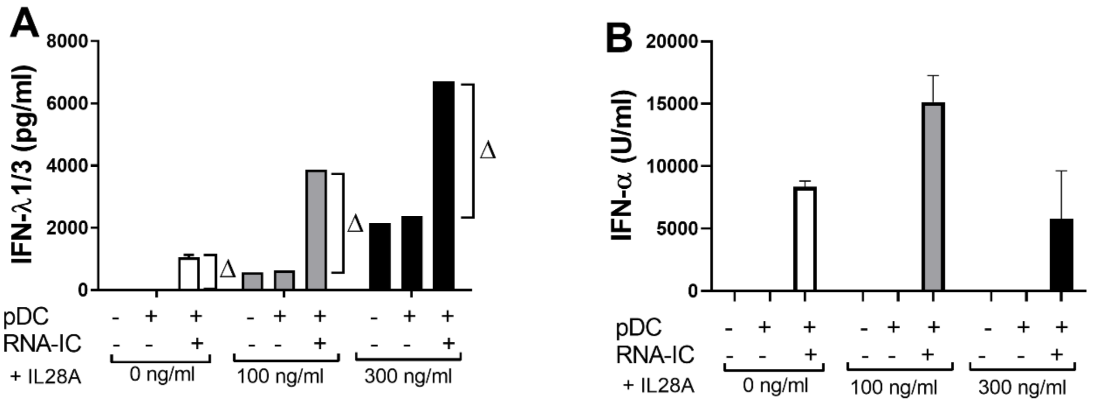

Additional file 1.

Figure S1. Interferon (IFN)-λ2 in cell supernatants displays crossreactivity with ELISA for Interferon (IFN)-λ1/3, but not with DELFIA for IFN-α

Measured protein levels of (A) IFN- λ1/3 or (B) IFN-α in medium, non-stimulated pDCs or RNA-IC-stimulated pDCs, supplemented with 0 ng/ml (white), 100 ng/ml (grey) or 300 ng/ml (black) IL28A. Bars show means with SD based on one of two representative donors.

The acquired IFN-λ1/3 values from priming experiments in supernatants from cells stimulated with RNA-IC and IFN-λ2 (figure 2A), were adjusted by subtracting the IFN-λ1/3 concentrations in IFN-λ2 supplemented medium, as indicated by a bracket and delta sign in the figure.
