# Supplementary material for: The regulation and pharmacological modulation of immune complex induced type III IFN production by plasmacytoid dendritic cells
Source: Arthritis Res Ther. 2020 Jun 5;22:130. doi: 10.1186/s13075-020-02186-z (PMC7275601; doi:10.1186/s13075-020-02186-z)
Supplement: Supplementary file 2 — Additional file 2: Methods. Gene expression microarray. Single-cell RNA expression profiling [file 13075_2020_2186_MOESM2_ESM.pdf]

Additional file S2. Methods

Gene expression microarray

50ng RNA per sample was used to produce Cy3-labeled cRNA. RNA samples were amplified and labeled using the Low Input Quick Amp Labeling Kit (Agilent Technologies). According to manufacturer's guidelines, 600 ng Cy3-labeled fragmented cRNA was hybridized 17 hours, at 65°C to Agilent Whole Human Genome Oligo Microarrays 8x60K, and the fluorescence signals of the hybridized probes were scanned. For readout and processing of the microarray image files, the Agilent Feature Extraction Software (FES) was used. To compare single intensity profiles in a ratio experiment, the Rosetta Resolver gene expression data analysis system (Rosetta Biosoftware) was used, ratios calculated by dividing sample signal intensity through control signal intensity.

Single-cell RNA expression profiling

Subsequently, a total of 1200 cells were encapsulated in droplets and barcoded sequencing libraries were prepared using the ddSEQ™ Single-Cell Isolator (Biorad) [23] and sequenced on an Illumina Hiseq 2500. Sequence data was transformed and demultiplexed using the "UMIs" (unique molecular identifier) command line tool [24] and the expression levels of genes per cell were estimated using Kallisto [25] with the GRCh38 gene set as reference. The automatic cell barcode cutoff calculation in UMIs was used to separate cells from background noise. With Seurat 3.0.1 [26] cell data was merged from the two donors and expression values normalized for donor specific expression, fraction of mitochondrial expression and read depth. The 2000 most variable genes were then used to for principal component analysis, which in turn was the base for Uniform Manifold Approximation and Projection (UMAP) dimensional reduction and non-supervised graph-based clustering. Differentially expressed genes between clusters were identified using Wilcoxon rank sum test.
