# Supplementary material for: The regulation and pharmacological modulation of immune complex induced type III IFN production by plasmacytoid dendritic cells
Source: Arthritis Res Ther. 2020 Jun 5;22:130. doi: 10.1186/s13075-020-02186-z (PMC7275601; doi:10.1186/s13075-020-02186-z)
Supplement: Supplementary file 3 — Additional file 3: Table S1. Clinical characteristics of SLE patients and their treatment at the time of blood sampling. [file 13075_2020_2186_MOESM3_ESM.pdf]

**Patient characteristics** (n=23)	
Females n (%)	20 (87)
Age years, median (range)	51 (32-81)
Disease duration years, median (range)	17.5 (1-46)
SLEDAI 2K* 0p	13 (57)
SLEDAI 2K 1-3p	3 (13)
SLEDAI 2K >4p	7 (30)
Anti-malarials n (%)	14 (61)
Mycophenolate mophetil n (%)	5 (20)
Azathioprine n (%)	6 (26)
Glucocorticoids n (%)	15 (65)
Glucocorticoid dose (mg) median (range)	5 (0-10)
Methotrexate n (%)	4 (16)
Cyclosporine	1 (4)
No immunosuppression n (%)	2 (9)

Additional file 3

Table S1. Clinical characteristics of SLE patients and their treatment at the time of blood sampling.

*SLEDAI 2K SLE Disease Activity Index 2000 [1]

1. Gladman DD, Ibanez D, Urowitz MB. Systemic lupus erythematosus disease activity index 2000. *J Rheumatol* 2002;29:288-91.
