# Supplementary material for: The regulation and pharmacological modulation of immune complex induced type III IFN production by plasmacytoid dendritic cells
Source: Arthritis Res Ther. 2020 Jun 5;22:130. doi: 10.1186/s13075-020-02186-z (PMC7275601; doi:10.1186/s13075-020-02186-z)
Supplement: Supplementary file 4 — Additional file 4: Figure S2. Type III IFN production is induced in pDCs and pDC-B cell co-cultures stimulated with RNA containing immune complexes (RNA-IC). [file 13075_2020_2186_MOESM4_ESM.pdf]

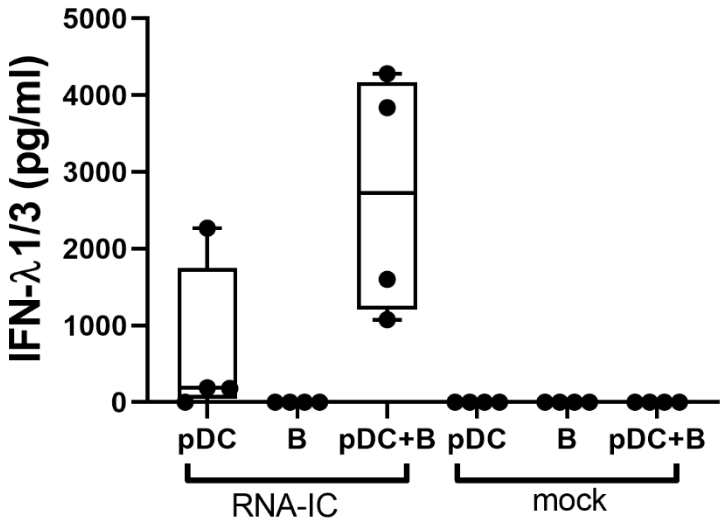

Additional file 4

Figure S2. Type III IFN production is induced in plasmacytoid dendritic cells (pDCs) and pDC-B cell co-cultures from healthy blood donors stimulated with RNA containing immune complexes (RNA-IC) Protein levels of IFN-λ1/3 in supernatants of pDC, B cells or pDC-B cell co-cultures after 20 h of RNA-IC or mock stimulation. Boxplots show medians with interquartile range, based on four healthy blood donors.
