# Supplementary material for: The regulation and pharmacological modulation of immune complex induced type III IFN production by plasmacytoid dendritic cells
Source: Arthritis Res Ther. 2020 Jun 5;22:130. doi: 10.1186/s13075-020-02186-z (PMC7275601; doi:10.1186/s13075-020-02186-z)
Supplement: Supplementary file 5 — Additional file 5: Figure S3. Type I IFN production is induced in pDCs and pDC-NK or pDC-B cell co-cultures stimulated with RNA-IC. [file 13075_2020_2186_MOESM5_ESM.pdf]

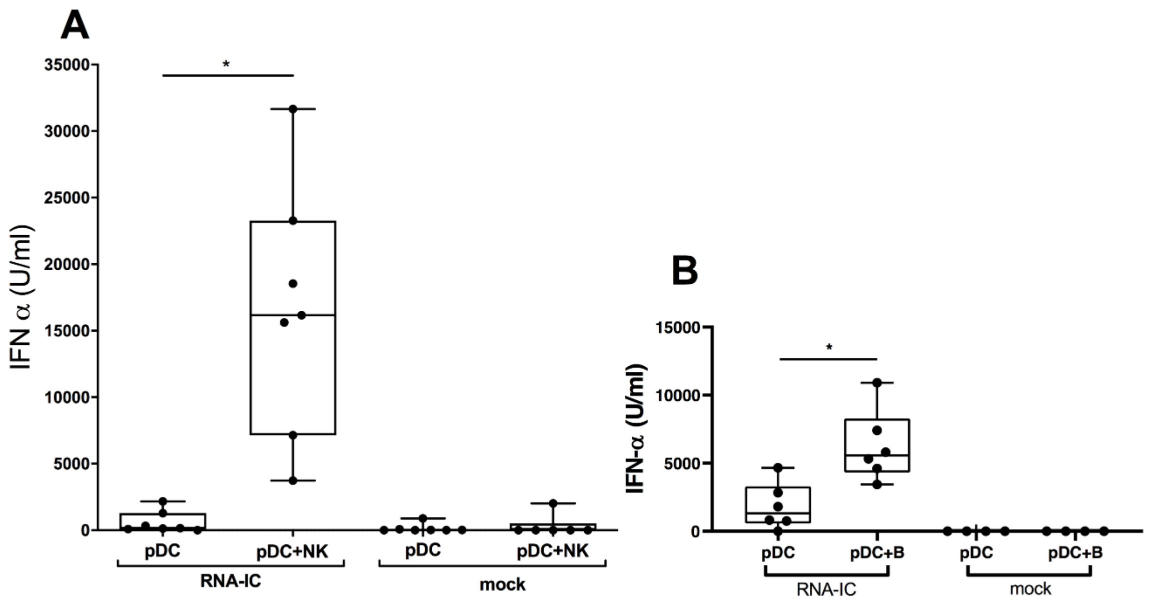

Additional file 5

Figure S3. Type I IFN production is induced in plasmacytoid dendritic cells (pDCs) and pDC-NK cell or pDC-B cell co-cultures from healthy blood donors stimulated with RNA containing immune complexes (RNA-IC). Levels of IFN-α in supernatants from (A) pDC and pDC-NK cells (B) pDC and pDC-B cells after 20 h of RNA-IC stimulation. No IFN-α was detected in pure NK or B cell cultures, not shown. Boxplots show medians with interquartile range for (A) seven donors (B) six donors. Wilcoxon´s test. *p<0.05.
