# Supplementary material for: The regulation and pharmacological modulation of immune complex induced type III IFN production by plasmacytoid dendritic cells
Source: Arthritis Res Ther. 2020 Jun 5;22:130. doi: 10.1186/s13075-020-02186-z (PMC7275601; doi:10.1186/s13075-020-02186-z)
Supplement: Supplementary file 6 — Additional file 6: Figure S4. Interleukin (IL)-3, IL-6, GM-CSF and interferon (IFN) –α increase type I IFN production by pDCs stimulated with RNA-IC. [file 13075_2020_2186_MOESM6_ESM.pdf]

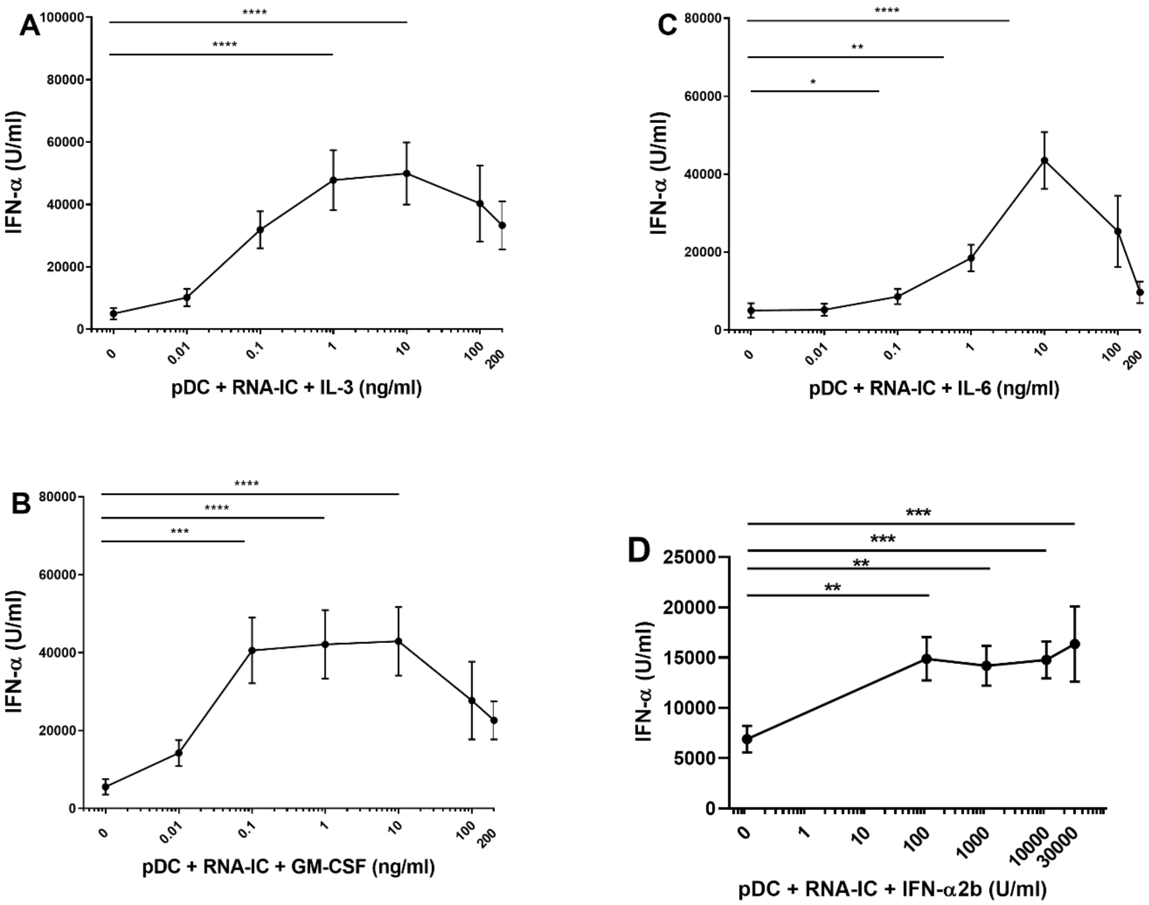

Additional file 6

Figure S4. Interleukin (IL)-3, IL-6, Granulocyte-macrophage colony stimulating factor (GM-CSF) and interferon (IFN)–α increase type I IFN production by plasmacytoid dendritic cells (pDCs) stimulated with RNA containing immune complexes (RNA-IC). The levels of IFN-α after 20h in supernatants from healthy donor pDCs cultivated in the presence of RNA-IC and (A) IL-3, (B) IL-6, (C) GM-CSF (D) IFN-α2b, in indicated concentrations. No IFN production was detected in the absence of RNA-IC, not shown. Graphs show means with SEM based on six donors in two independent experiments. Friedman´s test.*$p<0.05$, **$p<0.01$, ***$p<0.001$, ****$p<0.0001$.
