# Supplementary material for: The regulation and pharmacological modulation of immune complex induced type III IFN production by plasmacytoid dendritic cells
Source: Arthritis Res Ther. 2020 Jun 5;22:130. doi: 10.1186/s13075-020-02186-z (PMC7275601; doi:10.1186/s13075-020-02186-z)
Supplement: Supplementary file 7 — Additional file 7: Table S2. Differentially expressed genes (DEGs) in RNA-IC stimulated pDCs overexpressed in cluster “1” vs cluster “0”. 54 top overexpressed genes in cluster “1” compared to cluster “0”. [file 13075_2020_2186_MOESM7_ESM.pdf]

Additional file 7

Table S2 Differentially expressed genes (DEGs) in RNA-IC stimulated pDCs cluster "1" vs cluster "0" as following unsupervised clustering of the 2000 most variable genes. Lists DEGs (n=54) with adjusted p-value<E-15 (Bonferroni correction), sorted in declining order according to log2fold change (FC) of gene expression in cluster 1 compared to cluster 0.

gene_name	p_value	log2FC_cluster1_vs_0	cluster_1	cluster_0	adj_p_value	gene_description	gene_ID
IFNW1	5.67E-164	5.294	0.956	0.026	6.45E-160	interferon omega 1	HGNC:5448
IFNA2	1.64E-220	4.686	0.978	0.012	1.86E-216	interferon alpha 8	HGNC:5429
IFNB1	1.77E-195	4.242	0.956	0.017	2.01E-191	interferon beta 1	HGNC:5434
IFNA14	2.93E-220	3.823	0.889	0.007	3.33E-216	interferon alpha 5	HGNC:5426
IFNA10	1.41E-197	3.820	0.756	0.004	1.61E-193	interferon alpha 2	HGNC:5423
IFNA8	1.05E-183	3.777	0.756	0.007	1.19E-179	interferon alpha 21	HGNC:5424
IFNA6	1.05E-163	3.767	0.644	0.004	1.20E-159	interferon alpha 16	HGNC:5421
IFNA5	4.84E-225	3.682	0.844	0.004	5.50E-221	interferon alpha 14	HGNC:5420
IFNA1	3.16E-194	3.637	0.711	0.003	3.59E-190	interferon alpha 1	HGNC:5417
IFNA17	1.87E-202	3.436	0.756	0.004	2.13E-198	interferon alpha 7	HGNC:5428
CCL4	1.18E-124	3.136	0.822	0.028	1.34E-120	C-C motif chemokine ligand 4	HGNC:10630
IFNA4	8.02E-178	3.114	0.622	0.001	9.12E-174	interferon alpha 13	HGNC:5419
IFNA21	7.18E-198	3.102	0.667	0.001	8.15E-194	interferon alpha 10	HGNC:5418
IFNA16	1.05E-192	3.090	0.689	0.002	1.20E-188	interferon alpha 6	HGNC:5427
BCL2A1	9.23E-100	2.912	0.956	0.064	1.05E-95	BCL2 related protein A1	HGNC:991
CCL4L2	2.23E-122	2.742	0.756	0.022	2.53E-118	C-C motif chemokine ligand 4 like 2	HGNC:24066
CCL3	3.91E-132	2.336	0.556	0.005	4.44E-128	C-C motif chemokine ligand 3	HGNC:10627
IFIT2	3.44E-29	2.262	1	0.671	3.91E-25	interferon induced protein with tetratricopeptide repeats 2	HGNC:5409
PUS10	3.05E-87	2.247	0.622	0.023	3.47E-83	pseudouridine synthase 10	HGNC:26505
IFNA7	6.93E-116	2.206	0.4	0.001	7.87E-112	interferon alpha 17	HGNC:5422
BCAT1	8.88E-42	2.201	0.422	0.027	1.01E-37	branched chain amino acid transaminase 1	HGNC:976
TNF	1.01E-32	2.199	0.711	0.131	1.14E-28	tumor necrosis factor	HGNC:11892
CCL5	1.65E-24	2.122	0.289	0.023	1.88E-20	C-C motif chemokine ligand 5	HGNC:10632
NR4A3	6.83E-34	2.113	0.822	0.176	7.76E-30	nuclear receptor subfamily 4 group A member 3	HGNC:7982
IFNA13	1.12E-115	2.106	0.378	0	1.27E-111	interferon alpha 4	HGNC:5425
IFNL1	3.28E-75	2.094	0.244	0	3.73E-71	interferon lambda 1	HGNC:18363
CARMIL1	2.47E-52	2.016	0.711	0.07	2.80E-48	capping protein regulator and myosin 1 linker 1	HGNC:21581
ATF3	1.85E-59	1.982	0.578	0.035	2.10E-55	activating transcription factor 3	HGNC:785
TMEM267	1.07E-23	1.953	0.378	0.043	1.21E-19	transmembrane protein 267	HGNC:26139
PPP1R15A	1.21E-42	1.913	0.644	0.071	1.38E-38	protein phosphatase 1 regulatory subunit 15A	HGNC:14375
CD40	1.06E-32	1.896	0.822	0.184	1.20E-28	CD40 molecule	HGNC:11919
GPR34	2.37E-99	1.857	0.4	0.003	2.69E-95	G protein-coupled receptor 34	HGNC:4490
ID2	6.71E-29	1.846	0.689	0.127	7.62E-25	inhibitor of DNA binding 2	HGNC:5361
OASL	2.99E-23	1.828	0.867	0.374	3.40E-19	2'-5'-oligoadenylate synthetase like	HGNC:8090
RGS1	1.74E-25	1.747	0.489	0.069	1.97E-21	regulator of G protein signaling 1	HGNC:9991
TGIF1	9.92E-43	1.687	0.444	0.029	1.13E-38	TGFB induced factor homeobox 1	HGNC:11776
RNF144B	3.69E-25	1.683	0.778	0.194	4.19E-21	ring finger protein 144B	HGNC:21578
SLC7A11	6.16E-27	1.636	0.378	0.036	7.00E-23	solute carrier family 7 member 11	HGNC:11059
PMAIP1	1.06E-23	1.627	0.578	0.104	1.20E-19	phorbol-12-myristate-13-acetate-induced protein 1	HGNC:9108
REL	2.65E-22	1.603	0.867	0.289	3.01E-18	REL proto-oncogene, NF-kB subunit	HGNC:9954
ADAM32	7.06E-48	1.595	0.356	0.014	8.02E-44	ADAM metallopeptidase domain 32	HGNC:15479
ST8SIA4	3.88E-32	1.536	0.511	0.056	4.41E-28	ST8 alpha-N-acetyl-neuraminide alpha-2,8-sialyltransferase 4	HGNC:10871
DNAJB4	2.45E-21	1.513	0.667	0.148	2.78E-17	DnaJ heat shock protein family (Hsp40) member B4	HGNC:14886
BTN3A2	1.45E-23	1.508	0.978	0.548	1.64E-19	butyrophilin subfamily 3 member A2	HGNC:1139
MAP3K8	7.35E-32	1.492	0.311	0.019	8.35E-28	mitogen-activated protein kinase kinase kinase 8	HGNC:6860
TAL1	6.48E-45	1.475	0.222	0.004	7.37E-41	TAL bHLH transcription factor 1, erythroid differentiation factor	HGNC:11556
NFKBIZ	1.96E-20	1.455	0.444	0.069	2.23E-16	NFKB inhibitor zeta	HGNC:29805
FST	3.66E-42	1.412	0.156	0.001	4.16E-38	follistatin	HGNC:3971
CCL3L3	9.37E-49	1.374	0.178	0.001	1.06E-44	C-C motif chemokine ligand 3 like 3	HGNC:30554

CD83	8.12E-20	1.365	0.933	0.366	9.23E-16	CD83 molecule	HGNC:1703
ZFP36L1	1.29E-30	1.362	0.356	0.026	1.46E-26	ZFP36 ring finger protein like 1	HGNC:1107
NOMO2	1.63E-22	1.358	0.422	0.056	1.86E-18	NODAL modulator 2	HGNC:22652
IL12A	2.18E-48	1.097	0.156	0	2.48E-44	interleukin 12A	HGNC:5969
RANBP3L	8.73E-62	1.080	0.2	0	9.92E-58	RAN binding protein 3 like	HGNC:26353
