# Supplementary material for: The regulation and pharmacological modulation of immune complex induced type III IFN production by plasmacytoid dendritic cells
Source: Arthritis Res Ther. 2020 Jun 5;22:130. doi: 10.1186/s13075-020-02186-z (PMC7275601; doi:10.1186/s13075-020-02186-z)
Supplement: Supplementary file 8 — Additional file 8: Table S3. Differentially expressed genes (DEGs) in RNA-IC stimulated pDCs overexpressed in cluster “0” vs cluster “1” following unsupervised clustering of the 2000 most variable genes. [file 13075_2020_2186_MOESM8_ESM.pdf]

Additional file 8

Table S3 Differentially expressed genes (DEGs) in RNA-IC stimulated pDCs cluster "1" vs cluster "0" as following unsupervised clustering of the 2000 most variable genes. Lists the DEGs (n=19) overexpressed in cluster "0" compared to cluster "1" out of 164 DEGs with adjusted p-value <0.05 (Bonferroni correction), sorted in ascending order according to log2fold change (FC) of gene expression in cluster 1 compared to cluster 0.

gene_name	p_value	log2FC_cluster_1_vs_0	cluster_1	cluster_0	adj_p_value	gene_description	gene_ID
HERPUD1	1.21E-13	-1.570	0.289	0.833	1.38E-09	homocysteine inducible ER protein with ubiquitin like domain 1	HGNC:13744
SLFN5	5.22E-07	-1.262	0.111	0.517	5.93E-03	schlafen family member 5	HGNC:28286
TLR7	9.14E-07	-1.234	0.222	0.565	1.04E-02	toll like receptor 7	HGNC:15631
BTG1	6.52E-09	-1.084	0.511	0.838	7.41E-05	BTG anti-proliferation factor 1	HGNC:1130
TKFC	7.44E-07	-1.025	0.356	0.659	8.45E-03	triokinase and FMN cyclase	HGNC:24552
CTSC	8.84E-07	-0.924	0.778	0.887	1.00E-02	cathepsin C	HGNC:2528
STAT1	2.14E-06	-0.829	0.489	0.768	2.43E-02	signal transducer and activator of transcription 1	HGNC:11362
EIF2AK2	3.01E-06	-0.828	0.378	0.692	3.42E-02	eukaryotic translation initiation factor 2 alpha kinase 2	HGNC:9437
OAS1	2.64E-06	-0.786	0.489	0.751	3.00E-02	2'-5'-oligoadenylate synthetase 1	HGNC:8086
RPL13	2.53E-11	-0.711	0.867	0.963	2.87E-07	ribosomal protein L13	HGNC:10303
RPS10-NUDT3	1.15E-07	-0.710	0.644	0.865	1.30E-03	RPS10-NUDT3 readthrough	HGNC:49181
PLAC8	9.19E-07	-0.707	0.756	0.888	1.04E-02	placenta associated 8	HGNC:19254
RPS10	3.10E-06	-0.680	0.622	0.803	3.52E-02	ribosomal protein S10	HGNC:10383
RPL11	1.09E-08	-0.641	0.733	0.947	1.23E-04	ribosomal protein L11	HGNC:10301
RPS20	1.02E-06	-0.586	0.822	0.906	1.16E-02	ribosomal protein S20	HGNC:10405
RPLP1	3.94E-11	-0.579	0.956	0.98	4.47E-07	ribosomal protein lateral stalk subunit P1	HGNC:10372
RPS28	3.53E-07	-0.538	0.844	0.934	4.01E-03	ribosomal protein S28	HGNC:10418
RPL27A	7.62E-07	-0.536	0.911	0.927	8.66E-03	ribosomal protein L27a	HGNC:10329
RPS19	1.32E-06	-0.451	0.933	0.97	1.50E-02	ribosomal protein S19	HGNC:10402
