# Supplementary material for: The regulation and pharmacological modulation of immune complex induced type III IFN production by plasmacytoid dendritic cells
Source: Arthritis Res Ther. 2020 Jun 5;22:130. doi: 10.1186/s13075-020-02186-z (PMC7275601; doi:10.1186/s13075-020-02186-z)
Supplement: Supplementary file 9 — Additional file 9: Figure S5. Interferon (IFN)-α production is triggered by RNA containing immune complexes (RNA-IC) in immune cells from systemic lupus erythematosus patients (SLE) patients and healthy controls. [file 13075_2020_2186_MOESM9_ESM.pdf]

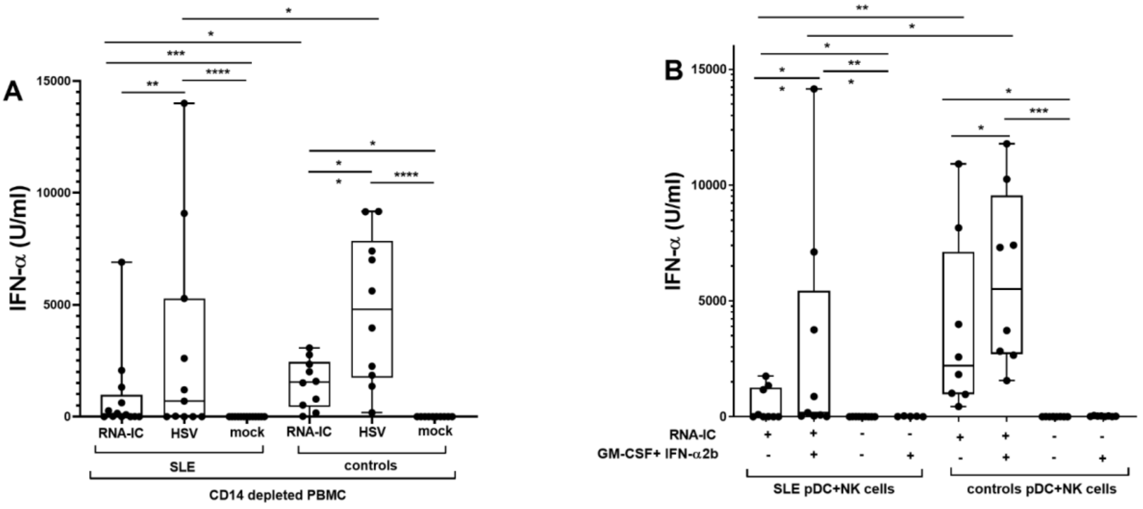

Additional file 9

Figure S5. Interferon (IFN)-α production is triggered by RNA containing immune complexes (RNA-IC) in immune cells from systemic lupus erythematosus patients (SLE) patients and healthy controls. The levels of IFN-α in supernatants after 20h by (A) monocyte depleted peripheral blood mononuclear cells (PBMC) in the presence of RNA-IC or Herpes simplex virus (HSV) and (B) plasmacytoid dendritic cell (pDC)-NK cell co-cultures with RNA-IC in the presence or absence of GM-CSF and IFN-α2b. Boxplots show medians with interquartile range, based on (A) 13 or (B) 10 patients and (A) 9 or (B) 8 healthy controls, respectively. Mann-Whitney test (SLE vs controls), Wilcoxon matched pairs signed rank test (RNA-IC vs HSV, priming vs no priming). *p<0.05, **p<0.01, ***p<0.001, ****p<0.0001.
